# Performance Evaluation of the Machine Learning Algorithms Used in Inference Mechanism of a Medical Decision Support System

**DOI:** 10.1155/2014/137896

**Published:** 2014-09-11

**Authors:** Mert Bal, M. Fatih Amasyali, Hayri Sever, Guven Kose, Ayse Demirhan

**Affiliations:** ^1^Department of Mathematical Engineering, Yildiz Technical University, Davutpasa Campus A-220, Esenler, 34220 Istanbul, Turkey; ^2^Department of Computer Engineering, Yildiz Technical University, Yildiz Campus, Besiktas, 34349 Istanbul, Turkey; ^3^Department of Computer Engineering, Hacettepe University, Beytepe Campus, Beytepe, 06530 Ankara, Turkey; ^4^Department of Information Management, Hacettepe University, Beytepe Campus, Beytepe, 06530 Ankara, Turkey; ^5^Department of Business Administration, Yildiz Technical University, Yildiz Campus, Besiktas, 34349 Istanbul, Turkey

## Abstract

The importance of the decision support systems is increasingly supporting the decision making process in cases of uncertainty and the lack of information and they are widely used in various fields like engineering, finance, medicine, and so forth, Medical decision support systems help the healthcare personnel to select optimal method during the treatment of the patients. Decision support systems are intelligent software systems that support decision makers on their decisions. The design of decision support systems consists of four main subjects called inference mechanism, knowledge-base, explanation module, and active memory. Inference mechanism constitutes the basis of decision support systems. There are various methods that can be used in these mechanisms approaches. Some of these methods are decision trees, artificial neural networks, statistical methods, rule-based methods, and so forth. In decision support systems, those methods can be used separately or a hybrid system, and also combination of those methods. In this study, synthetic data with 10, 100, 1000, and 2000 records have been produced to reflect the probabilities on the ALARM network. The accuracy of 11 machine learning methods for the inference mechanism of medical decision support system is compared on various data sets.

## 1. Introduction 

A decision support systems (DSSs) is a computer-based information system that supports organizational and business decision making activities. Medical decision support systems, which are variants of decision support systems, are intelligent software systems that are designed to improve clinical diagnosis system and to support the healthcare personnel in their decision. Intelligent decision support systems use artificial intelligence system techniques to support the healthcare personnel for selecting the best method for both diagnosis and also for treatment especially when the information about the treatment is incomplete or uncertain. These systems can work in both active and passive modes. When they are in passive mode, they will be used only when they are required. When they are in active mode, they will be making recommendations as well. When we look at the approaches of the inference mechanisms, which constitute the most important part of the medical decision support systems, these approaches can be divided into two parts such as rule-based systems and data-driven systems. Rule-based systems are constructed on the knowledge base, which are formed by if-then structures. In this structure, the information base is formed by the rules. The operation logic of the system is to find relevant rules on basis of the available information, operate them, and continue to search for a rule until a result has been obtained.

Those rule-based systems have some strong features as well as some disadvantages. For example, the performance of the system decreases and the maintenance of the system becomes difficult in case of the number of the rules being large enough. The examples of the medical decision support systems are MYCIN [1, 2], TRAUMAID [3], and RO^2^SE [4].

Data-driven systems, on the other hand, operate in large data stacks and support the decision making process using data mining methods. Several studies can be found on literature about data-driven systems. Some of these studies can be referred as Bayes networks [5], rough sets [6], and artificial neural networks [7] which are the examples of such studies. Data-driven systems are more flexible compared to the rule-based systems and they have the ability to learn by themselves.

In our previous study [8] ALARM network structure was used for the generated synthetic data on the same data set. When the results are examined in that study, it can be seen that the rule based method is more successful in the rate of 25% than the “Bayesian network based” method in all dimensions of the data sets. Besides, when both of these methods are combined and utilized together the success rate rises to 80%; that is, much higher rates are acquired in comparison to the values obtained by applying these methods individually.

In this study, the accuracy of 11 machine learning methods which can be used in the inference mechanism of the medical decision support systems is carried out on various data sets.

## 2. Decision Support Systems

Decision support systems (DSSs) are interactive computer-based systems or subsystems that are designed to help decision makers to decide and complete the decision process operations and also to determine and solve problems using communication technologies, information, documents, and models. They provide data storage and retrieval but enhance the traditional information access and retrieval functions with support for model building and model-based reasoning. They support framing, modeling, and problem solving. Typical application areas of DSSs are healthcare, management, and planning in business, the military, and any area in which management will encounter complex decision situations. DSSs are typically used for strategic and tactical decisions faced by upper-level management-decisions with a reasonably low frequency and high potential consequences, in which the time taken for thinking through and modeling the problem pays off generously in the long run [10].

Generally, decision support systems should include the following features.

- DSSs are used to support the decision making process not to accomplish operational processes.
- DSSs should support each phase of the decision making process.
- DSSs support the half or full configured decision environments.
- DSSs support each management levels from bottom to top.
- DSSs have interactive and user-friendly interfaces.
- DSSs use data and model as a basis.

**Figure 1:**
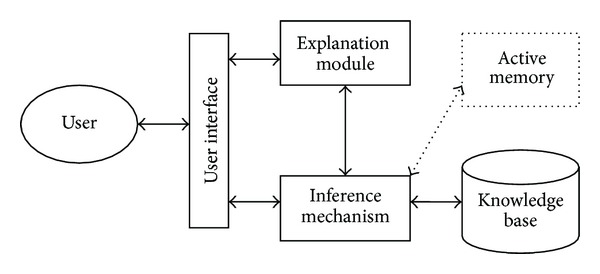
The main structure of decision support system.

Decision support systems and relevant operation methods can be divided into four main subjects. These subjects are called as inference mechanism, knowledge base, explanation module, and active memory. Inference mechanism constitutes the basis of decision support systems. In this part, the results are generated in consideration of the current information and/or the information that was entered to the system by the user. The generated results may be a decision or they may include guiding information. The second part is the knowledge base which holds the expert information used when the decision support system is making inference. The active memory part holds the information, which is supplied by the user and/or current inference processes. Also, explanation module, which may not be present on each decision support system, generates an accuracy validation and explanation in consideration of the results generated by the inference mechanism and knowledge base [11]. Those subjects and their relations are shown in Figure 1.

In rule-based systems, the knowledge base is formed by the rule group. The results are obtained for various circumstances on the problem relevant to the subject, using the generated rules. The rules forming the knowledge base are prepared by if-then structure. The content of an inference system, which is developed using rule-based methods, consists of the rules generated by if-then, the facts, and an interpreter that interprets the facts using the rules in the system [12].

There are two methods used to process the rules in the rule-based methods. These methods are forward chaining and backward chaining. In forward chaining method, the results are obtained using the preliminary facts with the help of the rules. In backward chaining method, it is started with a hypothesis (or target) and the rules, which will reach that hypothesis, are searched. The reached rules generate subrules and the process continues in this way.

In cases, which the result is estimated and this estimation should be verified, backward chaining method should be used instead of forward chaining method.

In order to generate the rule set in rule-based methods of inference systems, people who are experienced on the problem should contribute to the design of the system. This process usually proceeds with the help of experienced people in the rule development phase by determining the faults and defects in the estimations and using the planned system as a reference [13].

The designer usually develops simple interfaces for experts to contribute in the development phase. In the beginning of the process, the experts start testing the systems as if they will use the system for operational purposes. The questions asked to the experts in the scope of the limited information of the systems are answered by the same experts.

The aim is to test the system in order to improve it. The expert who answered the questions evaluates the system by looking at the results generated by the system and then tries to correct the defined defects and faults by using the rule development tool. The rule set in the inference systems, which use rule-based methods, can be generated by the expert on the problem.

Data-driven systems examine large data pools in organizations. These systems usually work with the systems that collect data like data warehouse, and so forth. Data-driven systems take place in decision making process with online analytical processing (OLAP) and data mining methods. These systems work on very large datasets. The relations in these datasets are analyzed electronically and make predictions for future data relations. Data-driven systems use the bottom-up procedure to explain the characteristics of the data system [14].

## 3. Machine Learning Algorithms

Machine learning is about learning to make predictions from example of desired behavior or past observations. Learning methods have found numerous applications in performance modeling and evaluation [15]. The basic definitions of machine learning are given below.

### 3.1. Basic Definitions

Data points called* examples* are typically described by their values on some set of* features.* The space that examples live in is called the* feature space* and is typically denoted by *X*.

The* label* of an example will be predicted. The space of possible labels is denoted by *Y*.

A* learning problem* is some unknown data distribution *D* over *X* × *Y*, coupled with a loss function *l*(*y*, *y*′) measuring the loss of predicting *y*′ when the true label is *y*.

A* learning algorithm* takes a set of labeled training examples of the form (*x*, *y*) ∈ *X* × *Y* and produces a predictor *f* : *X* → *Y*. The goal of the algorithm is to find *f* minimizing the expected loss ${\vec{E}}_{(x,y)\sim D}l\left(f\left(x\right),y\right)$.

There are two base learning problems, defined for any feature space *X*. In binary classification, examples are categorized into two categories [15].

**Definition 1 .**

A* binary classification* problem is defined by a distribution *D* over *X* × *Y*, where *Y* = {0,1}. The goal is to find a* classifier h* : *X* → *Y* minimizing the* error rate* on *D*:

(1) $$\begin{array}{c} e\left(h,D\right)={\vec{\mathrm{Pr}}}_{(x,y)\sim D}\left[h\left(x\right)\neq y\right]. \end{array}$$

By fixing an unlabeled example *x* ∈ *X*, a* conditional distribution D*∣*x* over *Y* is found.

Regression is another basic learning problem, where the goal is to predict a real-valued label *Y*.

The loss function typically used in regression is the squared error loss between the predicted and actual labels.

**Definition 2 .**

A* regression problem* is defined by a distribution *D* over *X* × *R*. The goal is to find a function *f* : *X* → *R* minimizing the* squared loss* [15]:

(2) $$\begin{array}{c} l\left(f,D\right)={\vec{E}}_{(x,y)\sim D}{\left(f\left(x\right)-y\right)}^{2}. \end{array}$$

The machine learning algorithms that are used in the study will be explained below.

### 3.2. C4.5 Decision Tree

A decision tree is basically a classifier that shows all possible outcomes and the paths leading to those outcomes in the form of a tree structure. Various algorithms for inducing a decision tree are described in existing literature, for example, CART (classification and regression tress) [16], OC1 [17], ID3, and C4.5 [18]. These algorithms build a decision tree recursively by partitioning the training data set into successively purer subsets [19].

C4.5 [18] is an algorithm used to generate a decision tree. C4.5 uses the fact that each attribute of the data can be used to make a decision that splits the data into smaller subsets. C4.5 examines the normalized information gain (difference in entropy) that results from choosing a feature for splitting the data [20]

(3) $$\begin{array}{c} \text{SplitInf}{\text{o}}_{x}T=-\overset{n}{\underset{i=1}{\sum }}\frac{T_{i}}{T}\text{lo}{\text{g}}_{2}\frac{T_{i}}{T},\\ \text{Gain}\;\text{Rati}{\text{o}}_{x}\left(T\right)=\frac{\text{Gai}{\text{n}}_{x}\left(T\right)}{\text{SplitInf}{\text{o}}_{x}T}, \end{array}$$

where SplitInfo_*x*_ *T* represents the potential information provided by dividing dataset, *T*, into *n* partition corresponding to the outputs of attributes *x*, and Gain_*x*_(*T*) is how much gain would be achieved by branching on *x*.

### 3.3. Multilayer Perceptron (MLP)

Multilayer perceptron (MLP) [21] also referred to as multilayer feed forward neural networks is the most used and popular neural network method. It belongs to the class of supervised neural network. The MLP topology consists of three sequential layers of processing nodes: an input layer, one or more hidden layers, and an output layer which produces the classification results.

**Figure 2:**
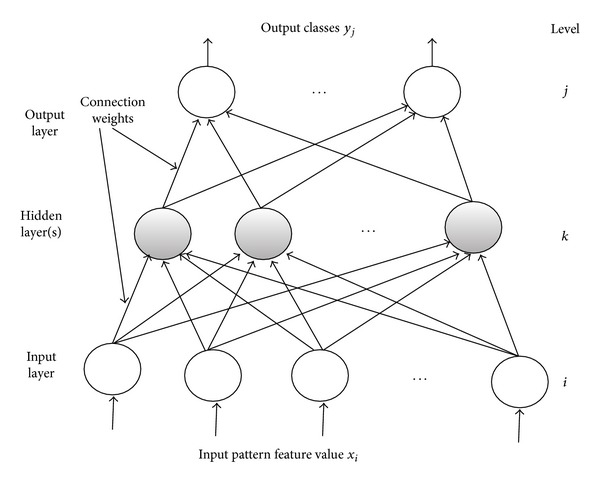
Structure of a multilayer perceptron [7].

A MLP structure is shown in Figure 2.

The principle of the network is that when data are presented at the input layer, the network nodes perform calculations in the successive layers until an output value is obtained at each of the output nodes. This output signal should be able to indicate the appropriate class for the input data. A node in MLP can be modeled as one or more artificial neurons, which computes the weighted sum of the inputs at the presence of the bias and passes this sum through the nonlinear activation function. This process is defined as follows [7]:

(4) $$\begin{array}{c} \mu_{j}=\overset{N}{\underset{i=1}{\sum }}w_{ji}x_{i}+\theta_{j},\\ y_{j}=\varphi_{j}\left(\mu_{j}\right), \end{array}$$

where *μ*_*j*_ is the linear combination of inputs *x*_1_, *x*_2_,…, *x*_*N*_, *θ*_*j*_ is the bias (adjustable parameter), *w*_*ji*_ is the connection synaptic weight between the input *x*_*i*_ and the neuron *j*, and *φ*(·) is the activation function (usually nonlinear function) of the *j*th neuron, and *y*_*j*_ is the output. Here, hyperbolic tangent and logistic sigmoid function can be used for the nonlinear activation function. But, in most of the applications widely used logistic sigmoid function is applied as follows:

(5) $$\begin{array}{c} \varphi \left(\lambda \right)=\frac{1}{1+e^{-\lambda }}, \end{array}$$

where *λ* represents the slope of the sigmoid [22].

The bias term *θ*_*j*_ contributes to the left or right shift of the sigmoid activation function, depending on whether *θ*_*j*_ takes a positive or negative value.

#### 3.3.1. Backpropagation Learning Algorithm

Learning in a MLP is an unconstrained optimization problem, which is subject to the minimization of a global error function depending on the synaptic weights of the network. For a given training data consisting of input-output patterns, values of synaptic weights in a MLP are iteratively updated by a learning algorithm to approximate the desired value. This update process is usually performed by backpropagating the error signal layer by layer and adapting synaptic weights with respect to the magnitude of error signal [23].

The first backpropagation learning algorithm for use with MLP structures was presented by [21]. The backpropagation algorithm is one of the simplest and most general methods for the supervised training of MLP. This algorithm uses a gradient descent search method to minimize a mean square error between the desired output and the actual outputs. Backpropagation algorithm is defined as follows [7, 24].

- Initialize all the connection weights *w* with small random values from a pseudorandom sequence generator.
- Repeat until convergence (either when the error *J* is below a preset value or until the gradient ∂*J*/∂*w* is smaller than a preset value).
  - Compute the update using Δ*w*(*m*) = −*ξ*(∂*J*(*m*)/∂*w*),
  - Iterative algorithm requires taking a weight vector at iteration *m* and updating it as *w*(*m* + 1) = *w*(*m*) + Δ*w*(*m*),
  - Compute the error *J*(*m* + 1),

where *m* is the iteration number, *w* represents all the weights in the network, and *ξ* is the learning rate and merely indicates the relative size of the change in weights. The error *J* can be chosen as the mean square error function between the actual output *y*_*j*_ and the desired output *d*_*j*_; *d* and *y* are the desired and the network output vector of length *N*:

(6) $$\begin{array}{c} J\left(w\right)=\frac{1}{2}\overset{N}{\underset{j=1}{\sum }}{\left(d_{j}-y_{j}\right)}^{2}=\frac{1}{2}{\left(d-y\right)}^{2}. \end{array}$$

### 3.4. Support Vector Machines (SVMs)

The support vector machines (SVMs) [25] is a type of learning machine based on statistical learning theory. SVMs are supervised learning methods that have been widely and successfully used for pattern recognition in different areas [26].

In particular in recent years SMVs with linear or nonlinear kernels have become one of the most promising learning algorithms for classification as well as regression [27]. The problem that SVMs try to solve is to find an optimal hyperplane that correctly classifies data points by separating the points of two classes as much as possible [28].

Let *x*_*i*_ (for 1 ≤ *i* ≤ *N*_*x*_) be the input vectors in input space, with corresponding binary labels *y*_*i*_ ∈ {−1,1}.

Let $\vec{\;X_{i}}=\Phi \left(x_{i}\right)$ be the corresponding vectors in feature space, where Φ(*x*_*i*_) is the implicit kernel mapping, and let *K*(*x*_*i*_, *x*_*j*_) = Φ(*x*_*i*_) · Φ(*x*_*j*_) be the kernel function, implying a dot product in the feature space [29].

*K*(*x*, *y*) represents the desired notion of similarity between data *x* and *y*. *K*(*x*, *y*) needs to satisfy a Mercer's condition in order for Φ to exist [28].

There are a number of kernel functions which have been found to provide good generalization capabilities [30].

The most commonly used kernel functions are as follows:

 Linear Kernel: *K*(*x*_*i*_, *x*_*j*_) = *x*_*i*_^*T*^ *x*_*j*_
 Polynomial Kernel: *K*(*x*_*i*_, *x*_*j*_) = (*η*(*x*_*i*_^*T*^ *x*_*j*_)+*r*)^*d*^
 Gaussian Kernel: *K*(*x*_*i*_, *x*_*j*_) = exp⁡(−*η*||*x*_*i*_−*x*_*j*_||^2^)
 Gaussian Radial Basis Function Kernel: *K*(*x*_*i*_, *x*_*j*_) = exp⁡(−*η*||*x*_*i*_−*x*_*j*_||^2^/2*σ* ^2^)
 Sigmoid Kernel: *K*(*x*_*i*_, *x*_*j*_) = tanh(*η*(*x*_*i*_ *x*_*j*_) + *r*)

where *η* > 0 and *r* are kernel parameters, *d* is the degree of kernel and positive integer number, and *σ* is the standard deviation and positive real number.

The optimization problem for a soft-margin SVM is

(7) $$\begin{array}{c} \underset{\vec{w},b}{\min}\left\{\frac{1}{2}{||\vec{w}||}^{2}+C\sum_{i}\xi_{i}\right\} \end{array}$$

subject to the constraints $y_{i}\left({\vec{w}}_{i}x+b\right)=1-\xi_{i}$ and *ξ*_*i*_ ≥ 0, where $\vec{w}$ is the normal vector of the separating hyperplane in feature space, and *C* > 0 is a regularization parameter controlling the penalty for misclassification. Equation (7) is referred to as the primal equation. From the Lagrangian form of (7), we derive the dual problem

(8) $$\begin{array}{c} \underset{\alpha }{\max}\left\{\sum_{i}\alpha_{i}-\frac{1}{2}\sum_{i,j}\alpha_{i}\alpha_{j}y_{i}y_{j}K\left(x_{i},x_{j}\right)\right\} \end{array}$$

subject to 0 ≤ *α*_*i*_ ≤ *C*. This is a quadratic optimization problem that can be solved efficiently using algorithms such as sequential minimal optimization (SMO) [31].

Typically, many *α*_*i*_ go to zero during optimization, and the remaining *x*_*i*_ corresponding to those *α*_*i*_ > 0 are called support vectors. To simplify notation, from here on we assume that all nonsupport-vectors have been removed, so that *N*_*x*_ is now the number of support vectors, and *α*_*i*_ > 0 for all *i*. With this formulation, the normal vector of the separating plane $\vec{w}$ is calculated as

(9) $$\begin{array}{c} \vec{w}=\overset{N_{x}}{\underset{i=1}{\sum }}\alpha_{i}y_{i}{\vec{x}}_{i}. \end{array}$$

Note that because $\vec{\;X_{i}}=\Phi \left(x_{i}\right)$ is defined implicitly, $\vec{w}$ exists only in feature space and cannot be computed directly. Instead, the classification $f\left(\vec{q}\right)$ of a new query vector $\vec{q}$ can only be determined by computing the kernel function of $\vec{q}$ with every support vector:

(10) $$\begin{array}{c} f\left(\vec{q}\right)=\mathrm{sign}\left(\overset{N_{x}}{\underset{i=1}{\sum }}\alpha_{i}y_{i}K\left(\vec{q},x_{i}\right)+b\right), \end{array}$$

where the bias term *b* is the offset of the hyperplane along its normal vector, determined during SVM training [29].

### 3.5. Naïve Bayes

Naïve-Bayes is one of the most efficient and effective inductive learning algorithms for machine learning and data mining [32].

**Figure 3:**
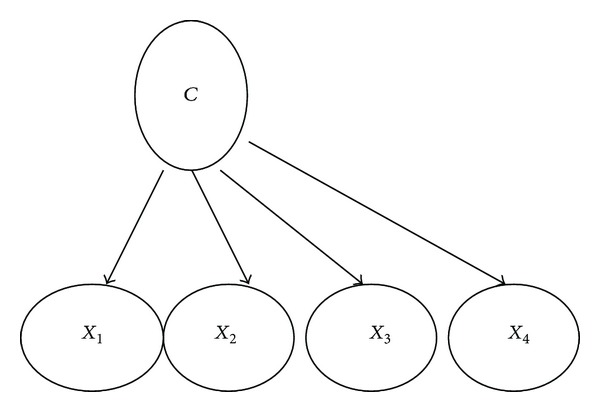
A simple Naïve-Bayes structure.

A Naïve-Bayes Bayesian network is a simple structure that has the classification node as the parent node of all other nodes. This structure is shown in Figure 3.

No other connections are allowed in a Naïve-Bayes structure. Naïve-Bayes has been used as effective classifier for many years. It has two advantages over many other classifiers. First, it is easy to construct, as the structure is given* a priori* (and hence no structure learning procedure is required). Second, the classification process is very efficient. Both advantages are due to its assumption that all the features are independent of each other. Although this independence assumption is obviously problematic, Naïve-Bayes has surprisingly outperformed many sophisticated classifiers over a large number of datasets, especially where the features are not strongly correlated [33].

The procedure of learning Naïve-Bayes (Figure 3) is as follows.

1. Let the classification node be the parent of all other nodes.
2. Learn the parameters (recall these are just the empirical frequency estimates) and output the Naïve-Bayes Bayesian network [34].

Typically, an example *E* is represented by a tuple of attribute values (*x*_1_, *x*_2_,…, *x*_*n*_), where *x*_*i*_ is the value of attribute *X*_*i*_. Let *C* represent the classification variable, and let *c* be the value of *C* [32]. Naïve-Bayes classifier is defined as below:

(11) classify(x1,x2,…,xn) =argmaxc⁡ p(C=c)∏i=1np(Xi=xi ∣ C=c).

### 3.6. Instance-Based Learning

Instance-based learning (IBL) [35] algorithms have several notable characteristics. They employ simple representations for concept descriptions, have low incremental learning costs, have small storage requirements, can produce concepts exemplars on demand, can learn continuous functions, and can learn nonlinearly separable categories; IBL algorithms have been successfully applied to many areas such as speech recognition, handwritten letter identification, and thyroid disease diagnosis.

All IBL algorithms consist of the following three components [36].

1. Similarity function: Given two normalized instances, this yields their numeric-valued similarity.
2. Classification function: Given an instance *i* to be classified and its similarity with each saved instance yields a classification for *i*.
3. Memory updating algorithm: Given the instance being classified and the results of the other two components updates the set of saved instances and their classification records.

The IB1 (one nearest neighbor) algorithm is the simplest instance-based learning algorithm. IB1 (one nearest neighbor) algorithm will be explained below.

#### 3.6.1. IB1 (One Nearest Neighbor)

IB1 [35] is an implementation of the simplest similarity based learner, known as nearest neighbor. IB1 simply finds the stored instance closest (according to Euclidean distance metric) to the instance to be classified. The new instance is assigned to the retrieved instance's class. Equation (12) shows the distance metric employed by IB1:

(12) $$\begin{array}{c} D\left(x,y\right)=\sqrt{\overset{n}{\underset{i=1}{\sum }}f\left(x_{i},y_{i}\right)}. \end{array}$$

Equation (10) gives the distance between two instances *x* and *y*; *x*_*i*_ and *y*_*i*_ refer to the *i*th feature value of instance *x* and *y*, respectively.

For numeric valued attributes*f*(*x*_*i*_, *y*_*i*_) = (*x*_*i*_−*y*_*i*_)^2^, for symbolic valued attributes *f*(*x*, *y*) = 0, if the feature values *x*_*i*_ and *y*_*i*_ are the same, and 1 if they differ [37].

### 3.7. Simple Logistic Regression

Logistic regressions are one of the most widely used techniques for solving binary classification problems. In the logistic regressions, the posterior probabilities *p*_i_*, *i* ∈ {1,2} are represented as in the following:

(13) $$\begin{array}{c} \Pi_{1}=\frac{\exp\left(\eta \right)}{1+\exp\left(\eta \right)},\;\;\Pi_{2}=1-\Pi_{1}, \end{array}$$

where *η* is a function of an input ${\vec{x}}_{0}$. For example, *η* is a linear function of the input ${\vec{x}}_{0}$, that is,

(14) $$\begin{array}{c} \eta ={\vec{\alpha }}^{T}{\vec{x}}_{0}+\beta \end{array}$$

and the parameters $\vec{\alpha },\beta$ are estimated by the maximum likelihood method.

*η* is an arbitrary function of ${\vec{x}}_{0}$. Note that if you choose an appropriate *η*, the model in (13) can represent some kinds of binary classification systems, such as neural networks and LogitBoost [38].

LogitBoost with simple regression functions as base learners is used for fitting the logistic models. The optimal number of LogitBoost iterations to perform is cross-validated, which leads to automatic attribute selection. This method is called “simple logistic” [39, 40]. LogitBoost algorithm is defined below.

#### 3.7.1. LogitBoost Algorithm

The LogitBoost algorithm [41] is based on the observation that AdaBoost [42] is in essence fitting an additive logistic regression model to the training data. An additive model is an approximation to a function

(15) $$\begin{array}{c} F\left(x\right)=\overset{N}{\underset{i=1}{\sum }}c_{i}f_{i}\left(x\right), \end{array}$$

where the *c*_*i*_ are constants to be determined and the *f*_*i*_ are basis functions. If it is assumed that *F*(*x*) is the mapping that is looked for to fit as our strong aggregate hypothesis and the *f*(*x*) are our weak hypothesis, then it can be shown that the two-class AdaBoost algorithm is fitting such a model by minimizing the criterion:

(16) $$\begin{array}{c} J\left(F\right)=E\left(\exp\left(-yF\left(x\right)\right)\right), \end{array}$$

where *y* is true class label in {−1,1}. LogitBoost minimizes this criterion by using Newton-like steps to fit an additive logistic regression model to directly optimize the binomial log-likelihood −log⁡(1 + exp⁡(−2*yF*(*x*))) [43].

### 3.8. Boosting

Boosting [44] is a meta-algorithm which can be viewed as a model averaging method. It is the most widely used ensemble method and one of the most powerful learning ideas introduced in the last twenty years. Originally designed for classification, it can also be profitably extended to regression. One first creates a “weak” classifier; that is, it suffices that its accuracy on the training set is only slightly better than random guessing. A succession of models is built iteratively, each one being trained on a dataset in which points misclassified (or, with regression, those poorly predicted) by the previous model are given more weight. Finally, all of the successive models are weighted according to their success and then the outputs are combined using voting (for classification) or averaging (for regression), thus creating a final model. The original boosting algorithm combined three weak learners to generate a strong learner [45].

#### 3.8.1. AdaBoost Algorithm

Let $\vec{X}=\left({\vec{x}}_{i},y_{i}\right)$, *i* = 1,2,…, *N* be a training sample of observations, where ${\vec{x}}_{i}\in R^{n}$ is an *n*-dimensional vector of features, and *y*_*i*_ is a binary label: *y*_*i*_ ∈ {−1, +1}.

In a practical situation the label *y*_*i*_ may be hidden, and the task is to estimate it using the vector of features. Let us consider the most simple linear decision function

(17) $$\begin{array}{c} u_{i}=u\left({\vec{x}}_{i}\right)=\overset{n}{\underset{j=0}{\sum }}w_{j}\cdot x_{ij}, \end{array}$$

where *x*_*i*0_ is a constant term.

A decision rule can be defined as a function of decision function and threshold parameter

(18) $$\begin{array}{c} f_{i}=f\left(u_{i},\Delta \right)=\{\begin{array}{cc} 1, & \text{if}\;u_{i}\geq \Delta ,\\ 0, & \text{otherwise}. \end{array} \end{array}$$

Let us consider minimizing the criterion

(19) $$\begin{array}{c} \overset{N}{\underset{i=1}{\sum }}\xi \left({\vec{x}}_{i},y_{i}\right)\exp\left(-y_{i}u\left({\vec{x}}_{i}\right)\right), \end{array}$$

where the weight function is given below:

(20) $$\begin{array}{c} \xi \left({\vec{x}}_{i},y_{i}\right):=\exp\left\{-y_{i}F\left({\vec{x}}_{i}\right)\right\}. \end{array}$$

It is assumed that the initial values of the ensemble decision function $F\left({\vec{x}}_{i}\right)$ are set to zero.

Advantages of the exponential compared with squared loss function were discussed in [46]. Unfortunately, it is not possible to optimize the step-size in the case of exponential target function. It is essential to maintain low value of the step size in order to ensure stability of the gradient-based optimization algorithm. As a consequence, the whole optimization process may be very slow and time-consuming. The AdaBoost algorithm was introduced in [42] in order to facilitate optimization process. The following Taylor-approximation is valid under assumption that values of $u\left({\vec{x}}_{i}\right)$ are small:

(21) $$\begin{array}{c} \exp\left\{-y_{i}u\left({\vec{x}}_{i}\right)\right\}\approx \frac{1}{2}\left[{\left(y_{i}-u\left({\vec{x}}_{i}\right)\right)}^{2}+1\right]. \end{array}$$

Therefore, quadratic-minimization (QM) model is applied in order to minimize (19).

Then, the value of the threshold parameters Δ for *u*_*i*_ is optimized and the corresponding decision rule *f*_*i*_ ∈ {−1, +1} is found.

Next, we will return to (19),

(22) $$\begin{array}{c} \overset{N}{\underset{i=1}{\sum }}\xi \left({\vec{x}}_{i},y_{i}\right)\exp\left(-cy_{i}f\left({\vec{x}}_{i}\right)\right), \end{array}$$

where the optimal value of the parameter *c* may be easily found:

(23) $$\begin{array}{c} c=\frac{1}{2}\log\left\{\frac{A}{B}\right\} \end{array}$$

and where

(24) $$\begin{array}{c} A=\sum_{y_{i}=f({\vec{x}}_{i})}\xi \left({\vec{x}}_{i},y_{i}\right),\;\;B=\sum_{y_{i}\neq f({\vec{x}}_{i})}\xi \left({\vec{x}}_{i},y_{i}\right). \end{array}$$

Finally, for the current boosting iteration, we update the function *F*

(25) $$\begin{array}{c} F_{\text{new}}\left({\vec{x}}_{i}\right)⟵F\left({\vec{x}}_{i}\right)+cf\left({\vec{x}}_{i}\right) \end{array}$$

and recomputed weight coefficients *ξ* according to (20) [47].

### 3.9. Bagging

Bagging [48] predictors is a method for generating multiple versions of a predictor and using these to get on aggregated predictor. The aggregation averages over the versions when predicting a numerical outcome and does a plurality vote when predicting a class. The multiple versions are formed by making bootstrap replicates of the learning set and using these as new learning sets. Tests on real and simulated data sets using classification and regression trees and subset selection in linear regression show that bagging can give substantial gains in accuracy. The vital element is the instability of the prediction method. If perturbing the learning set can cause significant changes in the predictor constructed, then bagging can improve accuracy [48].

### 3.10. Random Forest

Random forests [49] are a combination of tree predictors such that each tree depends on the values of a random vector sampled independently and with the same distribution for all trees in the forest. The generalization error of a forest of tree classifiers depends on the strength of the individual trees in the forest and the correlation between them. A random forest is a classifier consisting of a collection of tree-structured classifiers $\left\{h\left(\vec{x},\Theta_{k}\right),\;k=1,2,\ldots \right\}$, where the {Θ_*k*_} are independent identically distributed random vectors and each tree casts a unit vote for the most popular class at input $\vec{x}$.

### 3.11. Reduced Error Pruning Tree

Reduced error pruning (REP) was introduced by Quinlan [50], in the context of decision tree learning. It has subsequently been adapted to rule set learning as well [51]. REP produces an optimal pruning of a given tree, the smallest tree among those with minimal error with respect to a given set of* pruning examples* [51, 52]. The REP algorithm works in two phases: first the set of pruning examples *S* is classified using the given tree *T* to be pruned. Counters that keep track of the number of examples of each class passing through each node are updated simultaneously. In the second phase—a bottom-up pruning phase—those parts of the tree that can be removed without increasing the error of the remaining hypothesis are pruned away [53]. The pruning decisions are based on the node statistics calculated in the top-down classification phase.

### 3.12. ZeroR (Zero Rule)

Zero rule (ZeroR, 0-R) is a trivial classifier, but it gives a lower bound on the performance of a given a dataset which should be significantly improved by more complex classifiers. As such it is a reasonable test on how well the class can be predicted without considering the other attributes [54].

## 4. ALARM Network Structure and Datasets

In order to compare the performances (in terms of accuracy) of machine learning methods in the scope of this study, the network structure, which is used in scientific studies and known as ALARM (a logical alarm reduction mechanism) network [5] in literature is used. ALARM network is a network structure that is prepared by using real patient information for many variables and shows the probabilities derived from the real life circumstances. ALARM network calculates the probabilities for different diagnosis based on the current evidences and recently it has been used for many researchers. Totally there are 37 nodes in ALARM network and the relationships and conditional probabilities among these have been defined. The medical information has been coded in a graphical structure with 46 arches, 16 findings, and 13 intermediate variables that relate the examination results to the diagnosis problems that represent 8 diagnosis problems. Two algorithms have been applied to this Bayes network; one of them is a message-passing algorithm, developed by Pearl [55] to update the probabilities in the various linked networks using conditioning methods and the second one is that the exact inference algorithm, developed by Lauritzen and Spiegelhalter [56] for local probability calculations in the graphical structure. There are three variables named diagnosis, measurements, and intermediate variables in the ALARM network.

1. *Diagnosis* and the qualitative information are on the top of the network. Those variables do not belong to any predecessors and they are deemed mutually independent from the predecessors. Each node is linked to the particular and detailed value sets that represent the severity and the presence of a certain disease.
2. *Measurements* represent any current quantitative information. All continuous variables are represented categorically with a discrete interval set that divides the value set.
3. *Intermediate variables* show the element that can not be measured directly. The probabilities in the Bayes network can represent both objective and subjective information. ALARM network includes statistical data, logical conditional probabilities, which are calculated from the equations relevant to the variables, and a certain number of subjective valuations and it is usually used to form the network structure over synthetically data.

**Figure 4:**
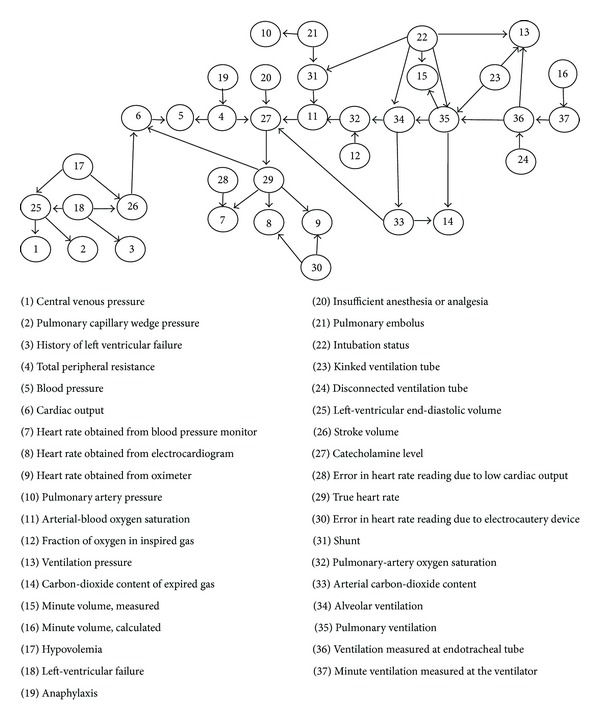
ALARM network structure and the variables defined in the network [9].

In cases for all given different predecessor nodes, it is required to obtain a conditional probability for a node. The structure of ALARM network and defined variable are shown in Figure 4.

**Figure 5:**
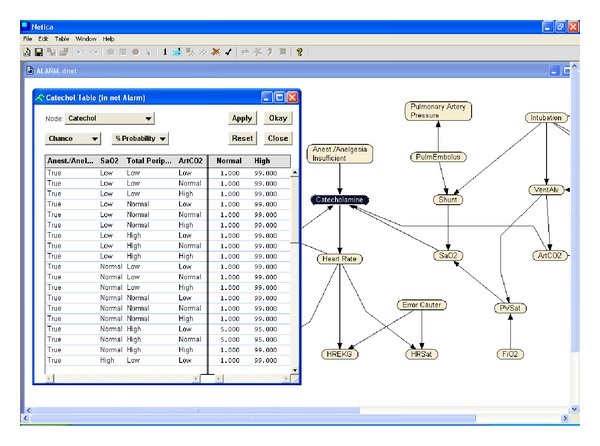
Conditional probability diagram for Alarm.dnet catechol variable.

In order to compare the performances of algorithms mentioned in Section 3, synthetic test data with 10, 100, 1000, and 2000 records have been produced to reflect the possibilities on the ALARM network. For these operations, based on ALARM network structure, NETICA 3.18 [57] software has been used. Conditional probability diagram for ALARM network structure and a variable defined in the structure are shown in Figure 5. Some of the synthetic data has been taken as test data.

Each record on those generated data shows probable values for each of the 37 variables that were defined on this network. Each record consist of values for intermediate variable as well as 12 input and 11 output variables. The tests, which were carried out, send the input variable values on each record to the relevant module and keep the resulting list as a separate file. The accuracy of the results is decided by comparing the variable values on the relevant record on the test data. For each record, 11 probable results have been obtained.

The results that were obtained by using JavaBayes [58] open source software are applied to each of the generated synthetic data sets separately. 11 output variables for one record belonging 100 data sets are shown in Table 1. JavaBayes uses a generalized version of “*variable elimination*” method as an inference algorithm [59]. It has generated 110 output variables in 10 data sets, 1100 output variables in 100 data sets, 11000 output variables in 1000 data sets, and 22000 output variables in 2000 data sets.

In Table 1, for each data set only 11 output variables for one record are presented. In this table, first column shows the variable name (disease name) and the second column shows the accuracy and they are calculated by the software using Bayes theorem, third column shows the real situations in the ALARM network, fourth column shows the results, generated by the software, and fifth column shows the comparison between the real situation and the results generated by the software. In the fifth column, if the real situation and the results generated by the software are the same POSITIVE and if the real situation and the results generated by the software are not the same NEGATIVE result will be generated. POSITIVE values show correct diagnosis, and NEGATIVE values show incorrect diagnosis.

For example, in Table 1, the accuracy of the MinVol variable has been calculated as 0.9136 by the software. Because this value is not the same with the real situation, the correct diagnosis has not been obtained. Similarly, for HREKG variable, the accuracy has been calculated as 0.8228 by the software. Because this value is the same with the real situation, the correct diagnosis has been obtained. Similar interpretations are also valid for other data sets. Each sample generated by ALARM network includes 12 independent and 11 depended variables. So we formed 11 classification datasets having 12 inputs and one output. The class labels for these 11 datasets are given at Table 2.

To see to effects of sample size, we generated several datasets having 10, 100, 1000, and 2000 samples for each of 11 classification datasets. At the end, we have 44 ( = 11∗4) classification datasets.

## 5. Experimental Design

We used 11 machine learning algorithms from WEKA library [60] for the classification of these 44 datasets. The algorithms are given in Table 3.

The default design parameters were selected for NB, MLP, SL, SMO, IBK, J48, and RT algorithms. For the meta-algorithms (boosting, bagging, and random forest) the ensemble sizes were selected as 100 to be sure from maximum accuracy.

## 6. Experimental Results

The performance of each classification algorithm was evaluated using 5 runs of 10-fold cross validation. In each 10-fold cross validation, each dataset is randomly split into 10 equal size segments and results are averaged over 50 (5∗10) trials. The classification results are divided by 4 according to the dataset's sample size. Tables 4, 5, 6, and 7 show the averaged classification accuracies with experiments having 10, 100, 1000, and 2000 samples, respectively.

**Figure 6:**
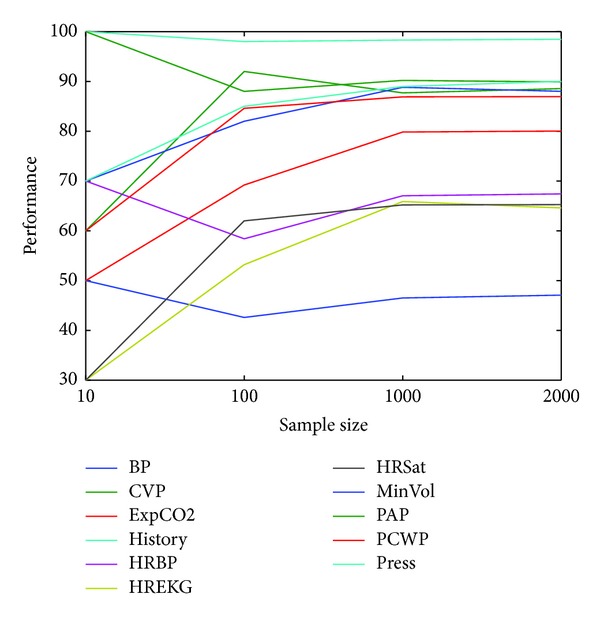
Classification accuracies changes with datasets' sample size.

Figure 6, shows the classification accuracies changes with the datasets' sample size. J48 decision tree is used as classifier in Figure 6.

As can be seen at Tables 4–7 and Figure 6 when the sample size increases it gives more accurate results, as expected. Zero rule defines accuracy by chance. It selects the most existent class for all samples. In BP and PAP datasets, none of the algorithms won the zero rule. This means that the datasets can not be learned by any of the algorithms.

We compared the accuracies of all classification algorithms in a pairwise manner in Table 8. To compare two algorithms' performances, we employed the statistically significance difference test (paired *t*-test) with 0.05 significance level. The win/loss records in Table 8 are the number of wins and losses of the algorithm in the row over the method in the column. The number of ties is the sum of wins and losses subtracted from 11. For example, J48 won over MLP on 5 datasets and the algorithms have similar performances on other 6 datasets. For the comparison, the datasets having 2000 samples were only used.

In addition to statistical difference test, we also compared the classification algorithms according to their average ranks. In the average rank comparison, for each of the datasets, the algorithms were ordered according to their performances. Then their ranks were averaged over 11 datasets. The average ranks and the sum of win and loses in Table 8 are given in Table 9.

According to Table 9, J48 (C4.5 decision tree) is the best ranked algorithm for our 11 datasets. The second one is bagging. According to the sum of wins, the best one is again J48.

To show the statistically meaningful difference between the average ranks we also applied the Nemenyi test [61]. According to is the Nemenyi test, the performance of two classifiers is significantly different if the corresponding average ranks differ by at least the critical difference (CD) calculated by

(26) $$\begin{array}{c} \text{CD}=q_{\alpha }\sqrt{\frac{k\left(k+1\right)}{6N}}. \end{array}$$

In (26), *k* is the number of classifiers compared, *N* is the number of datasets, *q*_*α*_ is the critical value, and *α* is the significance level. In our experiments, the critical value (*q*_0.05_) is 3.219 for 11 classifiers [62]. The critical difference (CD) is 3.129∗sqrt((11∗12)/(6∗11)) = 4.424. According to the Nemenyi test (at *P* < 0.05), there are no statistical differences between J48 and the algorithms having at most 4.424 + 3.55 = 7.974 average rank (NB, SL, SMO, BG, RF, and RT).

## 7. Conclusion

In cases of uncertainty and the lack of information, the most important part of the decision support systems which supports decision making process is the inference mechanism. There are data mining methods like SVM, MLP, decision trees, and so forth which are available in inference mechanism. Those methods can be used separately in an inference mechanism or also as a hybrid system, which consist of a combination of those methods.

In the study, for the generated synthetic data, ALARM network structure which is widely used in scientific studies has been used. This network structure is a structure that has been prepared using real patient information for many variables and shows the possibilities derived from the real life circumstances.

In this study, the performances of 11 machine learning algorithms (SVM, MLP, C4.5, etc.) are tested on 44 synthetic data sets (11 different dependent variables and 4 different dataset sizes). The comparison of algorithms we applied two different tests (statistically difference and average rank). C4.5 decision tree is the best algorithm according to the both of the tests for our 44 datasets. The datasets having more samples can be better predicted than having fewer samples.

In the future study, the comparison of the performances of the hybrid methods, which are combinations of the rule-based methods, and the data-driven methods and other machine learning systems will be carried out.

## Figures and Tables

**Table 1:** 11 Output variables for one record (100 datasets).

Variable name (disease)	Accuracy degree	Real situations	Results produced by the software	The comparison of the real situation and the result produced
History	0,9900	False	False	POSITIVE
Pres	0,9412	Normal	Zero	NEGATIVE
MinVol	0,9136	Normal	Zero	NEGATIVE
ExpCO2	0,9136	Normal	Zero	NEGATIVE
PAP	0,9000	Normal	Normal	POSITIVE
HRBP	0,8229	High	High	POSITIVE
HREKG	0,8229	High	High	POSITIVE
HRSat	0,8229	High	High	POSITIVE
CVP	0,7075	Normal	Normal	POSITIVE
PCWP	0,6970	Normal	Normal	POSITIVE
BP	0,4052	Low	Low	POSITIVE

**Table 2:** The class labels for 11 classification datasets.

Dependent variable (class)	Class labels
BP	Normal, low, high
CVP	Normal, low, high
ExpCO2	Normal, low, high, zero
History	False, true
HRBP	Normal, low, high
HREKG	Normal, low, high
HRSat	Normal, low, high
MinVol	Normal, low, high, zero
PAP	Normal, low, high
PCWP	Normal, low, high
Press	Normal, low, high, zero

**Table 3:** Used classification algorithms and abbreviations.

Algorithm name	Abbreviations
Zero rule	ZR
Naive-Bayes	NB
Multilayer perceptron	MLP
Simple logistic	SL
Support vector machines	SMO
One nearest neighbor	IBK
C4.5 decision tree	J48
Rep Tree	RT
Boosting	BS
Bagging	BG
Random forest	RF

**Table 4:** Classification accuracies with datasets having 10 samples (%).

	ZR	NB	MLP	SL	SMO	IBK	BS	BG	J48	RF	RT
BP	50.00	30.00	6.00	0.00	42.00	10.00	50.00	38.00	50.00	8.00	22.00
CVP	70.00	60.00	80.00	60.00	60.00	80.00	80.00	66.00	60.00	80.00	70.00
ExpCO2	50.00	50.00	60.00	50.00	52.00	50.00	50.00	46.00	50.00	54.00	20.00
History	100.00	100.00	100.00	100.00	100.00	100.00	100.00	100.00	100.00	100.00	100.00
HRBP	70.00	80.00	48.00	70.00	70.00	70.00	40.00	70.00	70.00	62.00	70.00
HREKG	60.00	70.00	42.00	48.00	60.00	40.00	30.00	58.00	30.00	44.00	60.00
HRSat	60.00	50.00	70.00	30.00	32.00	50.00	70.00	38.00	30.00	62.00	30.00
MinVol	70.00	70.00	80.00	70.00	72.00	70.00	80.00	70.00	70.00	72.00	70.00
PAP	100.00	100.00	100.00	100.00	100.00	100.00	100.00	100.00	100.00	100.00	100.00
PCWP	70.00	70.00	70.00	60.00	66.00	60.00	60.00	70.00	60.00	70.00	70.00
Press	70.00	70.00	80.00	70.00	70.00	70.00	80.00	70.00	70.00	70.00	70.00

**Table 5:** Classification accuracies with datasets having 100 samples (%).

	ZR	NB	MLP	SL	SMO	IBK	BS	BG	J48	RF	RT
BP	47.00	47.60	45.40	44.00	45.00	41.60	45.80	43.20	42.60	45.40	44.60
CVP	62.00	92.00	88.40	91.40	91.40	91.20	92.00	92.00	92.00	90.00	92.00
ExpCO2	65.00	70.80	73.20	74.00	69.20	69.00	65.00	67.60	69.20	71.60	66.20
History	98.00	98.00	100.00	98.00	98.00	98.00	100.00	98.00	98.00	98.40	98.00
HRBP	49.00	59.40	55.40	57.80	59.80	55.60	53.40	57.80	58.40	56.80	54.40
HREKG	48.00	55.60	56.80	54.80	56.80	54.20	54.00	50.80	53.20	54.60	50.20
HRSat	49.00	61.60	61.60	62.80	63.80	59.60	57.00	61.20	62.00	60.40	56.20
MinVol	79.00	84.00	86.20	84.60	85.20	82.00	79.00	79.00	82.00	83.40	79.00
PAP	88.00	88.00	86.80	88.00	88.00	88.00	88.00	88.00	88.00	87.40	88.00
PCWP	57.00	85.00	81.00	84.80	84.20	80.20	85.00	85.00	84.60	80.00	85.00
Press	80.00	85.00	89.80	87.20	85.20	84.00	80.40	82.20	85.00	85.60	81.40

**Table 6:** Classification accuracies with datasets having 1000 samples (%).

	ZR	NB	MLP	SL	SMO	IBK	BS	BG	J48	RF	RT
BP	45.00	46.32	44.52	46.44	46.58	44.58	45.00	47.18	46.52	44.84	46.60
CVP	67.70	87.64	87.00	87.72	87.08	87.14	85.60	87.70	87.70	87.06	87.68
ExpCO2	66.10	79.60	78.20	79.74	79.58	78.12	69.54	79.80	79.84	78.46	79.72
History	94.20	98.30	98.30	98.30	98.30	98.20	98.24	98.30	98.30	98.30	98.30
HRBP	48.00	67.12	66.18	67.20	67.56	66.48	59.20	67.06	67.04	66.62	66.88
HREKG	47.00	66.36	65.66	66.06	67.06	65.14	59.00	65.60	65.88	65.52	65.74
HRSat	47.40	65.68	65.02	65.88	66.84	64.60	59.00	65.14	65.20	64.68	65.10
MinVol	79.30	88.84	87.80	88.82	88.76	87.30	83.20	88.86	88.82	88.18	88.88
PAP	89.40	90.20	89.48	90.20	90.20	89.38	90.20	90.20	90.20	89.50	90.16
PCWP	65.20	86.80	86.40	86.90	86.72	86.18	83.80	86.90	86.90	86.50	86.90
Press	78.40	88.86	88.46	89.34	89.18	87.90	82.90	89.26	88.98	88.70	89.24

**Table 7:** Classification accuracies with datasets having 2000 samples (%).

	ZR	NB	MLP	SL	SMO	IBK	BS	BG	J48	RF	RT
BP	45.40	47.37	45.63	46.88	46.64	46.31	45.40	46.93	47.08	46.38	46.62
CVP	69.30	88.65	88.48	88.64	88.65	88.46	86.80	88.60	88.55	88.53	88.63
ExpCO2	66.65	80.15	79.19	80.25	80.23	78.96	70.24	79.98	80.03	79.42	79.99
History	94.15	98.45	98.40	98.43	98.45	98.12	98.42	98.45	98.45	98.43	98.45
HRBP	49.75	66.47	66.72	66.57	66.78	66.57	58.20	67.12	67.41	66.71	67.20
HREKG	48.80	65.26	63.92	65.00	64.85	64.79	57.25	64.91	64.60	65.10	64.52
HRSat	48.95	65.05	64.41	65.03	65.58	65.40	57.40	64.90	65.26	65.51	64.95
MinVol	77.70	88.02	87.44	87.99	87.93	86.91	82.00	88.04	88.01	87.59	88.03
PAP	88.95	89.90	89.43	89.88	89.86	89.38	89.90	89.90	89.90	89.53	89.90
PCWP	65.45	86.95	86.53	86.95	86.95	86.67	83.55	86.95	86.95	86.67	86.95
Press	78.20	89.68	89.62	90.03	90.07	89.27	82.80	90.11	90.00	89.73	90.00

**Table 8:** Pairwise comparison of accuracies (win/loss over 11 datasets) of all algorithms using 10 cv *t*-Test.

	ZR	NB	MLP	SL	SMO	IBK	BS	BG	J48	RF	RT
ZR	0/0	0/10	0/9	0/10	0/10	0/9	0/10	0/10	0/10	0/9	0/10
NB	10/0	0/0	3/0	0/0	0/0	5/0	8/0	0/0	0/1	3/0	0/0
MLP	9/0	0/3	0/0	0/3	0/3	2/0	8/1	0/4	0/5	0/0	0/4
SL	10/0	0/0	3/0	0/0	0/0	6/0	8/0	0/0	0/1	3/0	0/0
SMO	10/0	0/0	3/0	0/0	0/0	6/0	8/0	0/0	0/0	3/0	0/0
IBK	9/0	0/5	0/2	0/6	0/6	0/0	8/2	0/6	0/7	0/2	0/6
BS	10/0	0/8	1/8	0/8	0/8	2/8	0/0	0/8	0/8	1/8	0/8
BG	10/0	0/0	4/0	0/0	0/0	6/0	8/0	0/0	0/0	2/0	0/0
J48	10/0	1/0	5/0	1/0	0/0	7/0	8/0	0/0	0/0	4/0	0/0
RF	9/0	0/3	0/0	0/3	0/3	2/0	8/1	0/2	0/4	0/0	0/2
RT	10/0	0/0	4/0	0/0	0/0	6/0	8/0	0/0	0/0	2/0	0/0

**Table 9:** The average ranks of the algorithms over 11 datasets “and the sum of win/losses.”

Algorithm name	ZR	NB	MLP	SL	SMO	IBK	BS	BG	J48	RF	RT
Average rank	11	4.27	8.27	4.64	3.73	8	9.27	3.64	3.55	5.9	3.73
The number of wins/losses (over 110)	0/97	29/1	19/23	30/1	30/0	17/42	14/72	30/0	36/0	19/18	30/0
